# Clinical Cases

**Published:** 1890-01

**Authors:** G. W. Sherbino

**Affiliations:** Abilene, Texas


### CLINICAL CASES.

G. W. Sherbino, M. D., Abilene, Texas.

Religious Mania.—Mr. X., æt. twenty-one. Had been working hard on a farm. Took to reading the Bible excessively; would sit up late at night after a hard day's work to read.

He soon imagined he was called to preach, which he did do. He became sleepless; wanted to talk all the time on the Bible; he was very loquacious. I gave him Stramonium CMM, he seemed a little better.

They put him in jail for safe-keeping. He would strip off all his clothing every night, notwithstanding it was winter, compelling the jailer to go in and admonish him to dress himself. I gave him Hyos. CM. He improved so much that they took him home again. In a few days the father called at the office with his head and face scratched. He said his boy was perfectly wild; that he had tried to smash the clock, and when the father interfered he attempted to bite and strike. I sent Bell. CM (Skinner), which fixed him up in good shape. They sent him to the asylum for a short time, but he was perfectly quiet and rational.

